# Comparing intentional and unintentional poisoning among patients presenting to the emergency department at a tertiary care center in Lebanon: A retrospective descriptive analysis

**DOI:** 10.1371/journal.pone.0342694

**Published:** 2026-02-23

**Authors:** Alondra Barakat, Youssef Salam, Nadim Oueidat, Zyad Saifi, Hani Tamim, Ziad Kazzi, Eveline Hitti, Tharwat El Zahran

**Affiliations:** 1 Department of Emergency Medicine, American University of Beirut Medical Centre, Beirut, Lebanon; 2 Department of Internal Medicine, American University of Beirut Medical Centre, Beirut, Lebanon; 3 College of Medicine, Alfaisal University, Riyadh, Saudi Arabia; 4 Emory University, Atlanta, Georgia, United States of America; RMIT University, AUSTRALIA

## Abstract

**Introduction:**

Poisoning is a significant global public health issue, contributing substantially to emergency department (ED) visits. In 2021, the WHO reported nearly 2 million deaths and 53 million disability-adjusted life-years (DALYs) lost due to chemical exposures. Although regional data exist, Lebanon lacks national statistics on intentional and unintentional poisonings. This study compares these exposures in terms of patient characteristics, exposure agents, ED management, outcomes, and disposition.

**Methods:**

A retrospective chart review was performed using a toxicology database at a tertiary care center in Beirut from March 1, 2015, to August 1, 2023. All patients presenting with poisoning were included. Variables collected included demographics, psychiatric history, exposure route and type, ED course, and clinical outcomes.

**Results:**

Of 886 cases, 56.2% were intentional and 43.8% unintentional. Females predominated in intentional cases (68.3% vs. 47.9%, p < 0.001). Children under 6 years accounted for most unintentional exposures (0% vs. 48.5%, p < 0.001), while adults aged 20–59 were more often involved in intentional poisonings (69.9% vs. 31.2%, p < 0.001). Intentional exposures commonly involved sedatives, hypnotics, and antipsychotics (32.1%), while unintentional ones included analgesics (8%) and cleaning agents (7.5%). Mood disorders were reported in 45.6% of intentional cases. More patients were discharged from the ED in the unintentional group (50% vs. 38.4%, p < 0.001). Minor effects predominated in intentional exposures; unintentional cases more often showed no clinical effects. Moderate and severe outcomes occurred at similar rates.

**Discussion:**

This study reveals a higher prevalence of intentional poisoning in Lebanon, particularly among females and individuals with psychiatric disorders. Unintentional poisonings predominantly affected children under six, often due to poor storage and lack of child-resistant packaging. The findings underscore the need for regulatory policies, public education, and psychiatric support.

**Conclusion:**

Key differences between intentional and unintentional poisonings in Lebanon highlight the need for targeted preventive strategies and risk mitigation in high-risk populations.

## Introduction

Unintentional and intentional toxicological exposures remain major causes of morbidity and mortality across all age groups. In 2021, the WHO reported nearly 2 million deaths and 53 million disability-adjusted life-years (DALYs) lost due to chemical exposures, a composite measure combining years of life lost due to premature mortality and years lived with disability [1]. In 2022, America’s Poison Centers reported that 76.4% of cases were unintentional (accidental) poisoning, including general (45.1%), therapeutic error (16.1%), and misuse (8.57%). Intentional poisoning accounted for 18.9% of cases, with suicide as the leading cause (13.3%) [2]. The National Poison Data System (NPDS) recorded over 3,200 fatal poisonings, of which 80.6% were directly caused by the exposure, with 76% attributed to intentional poisoning. The most common exposures included analgesics, household cleaning substances, antidepressants, and cosmetics/personal care products [2]. This highlights the need for research and intervention strategies to address the complex challenges posed by drug poisoning [3].

While many developed countries have reliable reporting systems, many developing nations lack functional national poison centers, leading to significant underreporting. Over 90% of unintentional poisoning-related fatalities occur in lower-middle-income countries, with children disproportionately affected. Contributing factors include socioeconomic disparities, access to new opioids, psychotropics, over-the-counter drugs, domestic fuels, and hydrocarbons [1]. The Global Burden of Disease study highlights the limited data on toxic exposure in the Middle East due to underreporting and lack of awareness [2].

In Lebanon, limited research on poisoning underscores the need for comprehensive evaluation. Hitti et al. found that adult women and young children (under 5) constitute a significant portion of toxicological exposure cases in Lebanon [4]. Zahran et al. reported that 37% of pediatric poisoning ED visits were unnecessary, and 45% of unintentional poisoning cases were avoidable [5,6]. Assessing intentional vs unintentional poisoning is crucial for implementing effective healthcare interventions. This study compares the rates of intentional to unintentional poisoning at a tertiary care center in Lebanon and compares the findings with regional and global data.

## Methods

### Study design and setting

This retrospective cohort study was conducted at the American University of Beirut Medical Center (AUBMC), a tertiary care institution in Beirut, Lebanon. The study included patients treated for poisoning incidents between March 1, 2015, and August 1, 2023. The AUBMC Institutional Review Board (IRB) approved the study (protocol number: BIO-2023–0211), and patient consent was waived due to its retrospective design.

### Participants

The study included adult and pediatric patients who presented to the emergency department (ED) for poisoning during the study period. Poisoning was identified based on chief complaints and clinical assessments by attending physicians.

### Data collection and management

Charts were reviewed using REDCap (Research Electronic Data Capture), a secure web-based platform for managing research databases. REDCap is used as a tool for data entry, auditing, and seamless data export [7]. Patients were categorized into intentional and unintentional overdose groups according to the National Poison Data System (NPDS) standardized reason-for-exposure categories defined by the American Association of Poison Control Centers (AAPCC)—namely: Unintentional, Intentional (with subcategories such as suspected suicide, misuse, abuse), Adverse Reaction, Other, and Unknown—as specified in the NPDS Coding Users’ Manual [2]. Data collected included demographic information (age, gender), psychiatric history, exposure route, substance formulation, time from exposure to ED presentation, ED stay duration, decontamination methods, antidotes administered, and ED disposition. Outcomes were classified using the Medical Outcome Classification System from NPDS. NPDS is a database adapted from America’s Poison Centers that collects data from poison control centers across the United States [2,8].

### Statistical analysis

Statistical analysis was performed using IBM SPSS Statistics for Windows, Version 28.0. Descriptive statistics were used to summarize the dataset. Categorical variables were reported as frequencies and percentages, and continuous variables as means and standard deviations or as medians and interquartile ranges, as appropriate. The Shapiro–Wilk test was used to assess normality and to identify highly skewed, non-normal variables.Bivariate analysis examined categorical variables using the Chi-square test and continuous variables using the t-test. Multivariate logistic regression identified predictors of intentional poisoning. The variables were selected based on both statistical and clinical rationales. A p-value of 0.05 was set for the entry of potential predictors into the model, whereas a p-value of 0.1 was set for removal from the model. Due to the wide range of ages, we created a new age variable reflecting each 10-year increase. The results are presented as odds ratios (OR) and 95% confidence intervals (CI), with statistical significance set at *p* < 0.05.

## Results

Out of 886 poisoning cases, 498 (56.21%) were intentional, and 388 (43.79%) were unintentional. Most patients presented to the AUBMC ED, while the remaining cases were managed via telephonic consultation with the toxicology service team at AUBMC. Females predominated in the intentional poisoning group (intentional vs unintentional: 68.27% vs 46.94%; *p* < 0.001). Among children under 6 years, most poisonings were unintentional (intentional vs unintentional: 0% vs 48.45%; *p* < 0.001), whereas for patients aged 20–59 years, most cases were intentional (intentional vs unintentional: 69.88% vs 31.19%; *p* < 0.001).

In terms of psychiatric illnesses, a higher prevalence of mood disorders was noted in the intentional poisoning group (intentional vs unintentional: 66.87% vs 7.22%; *p* < 0.001), with mood disorders being more common (intentional vs unintentional: 45.58% vs 2.58%; *p* < 0.001). The intentional group also had a higher incidence of neurotic, stress-related, and somatoform disorders, including panic disorder, generalized anxiety disorder, social anxiety disorder, obsessive compulsive disorder (OCD), post-traumatic stress disorder (PTSD), and adjustment disorder (17.67% vs 3.87%; *p* < 0.001). Additionally, disorders related to substance abuse, childhood behavioral disorders, maltreatment, and eating disorders were more prevalent in the intentional poisoning group (intentional vs unintentional: 12.65% vs 0%; *p* < 0.001). Most unintentional poisoning cases had no psychiatric history (intentional vs unintentional: 22.49% vs 82.73%; *p* < 0.001).

Ingestion was the most common exposure route for both groups (intentional vs unintentional: 97.79% vs 54.12%; *p* < 0.001), followed by inhalation or nasal exposure (intentional vs unintentional: 2.21% vs 23.2%; *p* < 0.001). Dermal and parenteral exposures were more common in the unintentional group (dermal: 0.2% vs 4.64%; *p* < 0.001, parenteral: 0.8% vs 16.24%; *p* < 0.001).

Solid formulations were most common in both groups but more frequent in the intentional poisoning group (intentional vs unintentional: 91.57% vs 38.66%; *p* < 0.001). Additional details on baseline characteristics are in Table 1.

**Table 1:** Associations between baseline characteristics of patients with intentional and unintentional poisoning (*n* = 886).

Variables		Intentional poisoning (*n* = 498, 56.2%)	Unintentional poisoning (*n* = 388, 43.8%)	*p*-value
**Female**		340 (68.3%)	186 (47.9%)	**<0.001**
**Age (years)**	**Mean ± SD**	0.5 ± 14.7	19.2 ± 22.1	**<0.001**
	**Median (IQR)**	26.0 (20.0 - 38.0)	7.0 (2.0 - 32.0	
	**<6 years**	0 (0.0%)	188 (48.5%)	**<0.001**
	**6-12 years**	6 (1.2%)	27 (7.0%)	
	**13-19 years**	116 (23.3%)	22 (5.7%)	
	**20-59 years**	348 (69.9%)	121 (31.2%)	
	**>60 years**	28 (5.6%)	30 (7.7%)	
**Past psychiatric illnesses**		333 (66.9%)	28 (7.2%)	**<0.001**
**Mood disorders**		227 (45.6%)	10 (2.6%)	**<0.001**
**Neurotic/stress-related/somatoform disorders**		88 (17.7%)	15 (3.9%)	**<0.001**
**Disorders of adult personality and behavior**		61 (12.2%)	0 (0.0%)	**<0.001**
**Mental and behavioral disorders due to psychoactive substance abuse**		43 (8.6%)	0 (0.0%)	**<0.001**
**Schizophrenia/schizotypal/delusional disorder**		6 (1.2%)	2 (0.5%)	0.48
**Behavioral and emotional disorders with onset usually occurring in childhood and adolescence**		14 (2.8%)	2 (0.5%)	0.01
**Personal harm**		63 (12.6%)	0 (0.0%)	**<0.001**
**No psychiatric illness**		112 (22.5%)	321 (82.7%)	**<0.001**
Other*		88 (17.7%)	41 (10.6%)	0.003
**Route of exposure**				
**Ingestion**		487 (97.8%)	210 (54.1%)	**<0.001**
**Inhalation/nasal**		11 (2.2%)	90 (23.2%)	**<0.001**
**Dermal/Bite/sting**		3 (0.6%)	69 (17.8%)	**<0.001**
**Parenteral**		3 (0.6%)	16 (4.1%)	**<0.001**
**Ocular/Other**		3 (0.6%)	6 (1.5%)	0.19
**Substance formulation**				
**Solid (tablets/capsules/caplets)**		456 (91.6%)	150 (38.7%)	**<0.001**
**Liquid**		28 (5.6%)	132 (34.0%)	**<0.001**
**Aerosol/mist/spray/gas**		2 (0.4%)	33 (8.5%)	**<0.001**
**Powder granules/Cream/lotion/gel/ Food/other**		11 (2.2%)	19 (4.9%)	0.03
**Snake/ Insect**		1 (0.2%)	46 (11.9%)	**<0.001**
**Plant/ Mushroom**		0 (0.0%)	8 (2.1%)	0.001

* Behavioral syndromes associated with physiological disorders and physical factors; Behavioral and emotional disorders with onset usually occurring in childhood and adolescence; Maltreatment syndromes; Eating disorders; Unknown psychiatric illness.

Exposure to pesticides and cleaning substances was significantly more common in the unintentional poisoning group (pesticides: 1.41% vs 5.67%; *p* < 0.001, cleaning substances: 1.2% vs 7.47%; *p* < 0.001). Sedatives, hypnotics, and antipsychotics were more common in the intentional group (32.13% vs 6.44%; *p* < 0.001), with benzodiazepines like alprazolam more frequently used in intentional poisonings (7.23% vs 1.29%; *p* < 0.001). Analgesics and anti-inflammatory drugs were the second most common substances in the intentional poisoning group (intentional vs unintentional: 27.11% vs 7.99%; *p* < 0.001). Among them, acetaminophen is the most prevalent drug (20.28% intentional vs. 4.9% unintentional; *p* < 0.001). Opioids like codeine and tramadol were also common in the intentional group (5.02% vs 0%; *p* < 0.001).

Additionally, stimulants or “street drugs” like cannabis, cocaine, gamma-hydroxybutyrate (GHB), 3,4-methylenedioxy methamphetamine (MDMA), and caffeine are more commonly seen in intentional poisoning cases (intentional vs unintentional: 5.82% intentional vs. 0.77% unintentional; *p* < 0.001). More information about other substances involved in poison exposures is provided in Table 2.

**Table 2:** Associations between pharmacological/substance exposure and single agents involved in poison exposures with intentional and unintentional poisoning (*n* = 886).

Variables	Intentional poisoning (*n* = 498, 56.2%)	Unintentional poisoning (*n* = 388, 43.8%)	*p*-value
**Pesticides/insecticides**	7 (1.4%)	22 (5.7%)	**<0.001**
**Analgesic/anti-inflammatory**	135 (27.1%)	31 (8.0%)	**<0.001**
**Opioids**	25 (5.0%)	0 (0.0%)	**<0.001**
**Antidepressants**	111 (22.3%)	15 (3.9%)	**<0.001**
**Antibacterial/antifungal/antiviral/antiTB**	6 (1.2%)	3 (0.8%)	0.74
**Antihistamines**	13 (2.6%)	1 (0.3%)	**0.005**
**Decongestants**	1 (0.2%)	1 (0.3%)	1.00
**Cardiovascular drugs/antihypertensives**	25 (5.0%)	15 (3.9%)	0.41
**Anticonvulsants**	40 (8.0%)	2 (0.5%)	**<0.001**
**Sedatives/hypnotics/antipsychotics**	160 (32.1%)	25 (6.4%)	**<0.001**
**Cleaning substances**	6 (1.2%)	29 (7.5%)	**<0.001**
**Stimulants/street drugs**	29 (5.8%)	3 (0.8%)	**<0.001**
**Ethanol**	12 (2.4%)	9 (2.3%)	0.93
**Plants/herbs**	4 (0.8%)	9 (2.3%)	0.06
**Animals or insects (ingestion/bite/sting)**	2 (0.4%)	51 (13.1%)	**<0.001**
**Anticholinergic**	10 (2.0%)	9 (2.3%)	0.75
**Hormones and hormones antagonists/ Vitamins/minerals/Other substances**	34 (6.8%)	59 (15.2%)	**<0.001**

The management and disposition of these patients in the ED are shown in Table 3. The time between exposure and ED presentation is significantly longer for patients with intentional poisoning compared to those with unintentional poisoning (Mean ± SD in hours: 5.1 ± 14.6 vs 2.8 ± 3.7; *p* = 0.002). Moreover, patients with intentional poisoning also had longer stays in the ED compared to those with unintentional poisoning (Mean ± SD (in hours): 11.3 ± 11.3 vs 4.3 ± 5.2; *p* < 0.001). None of the unintentional poisoning cases received any decontamination method (intentional vs unintentional: 80.92% vs 100%; *p* < 0.001). In the intentional poisoning group, some patients received a single dose of activated charcoal (15.06% intentional vs 0% unintentional; *p* < 0.001), while others underwent nasogastric suction (2.21% intentional vs 0% unintentional; *p* = 0.003). Most unintentional poisoning cases did not receive any antidotes (96.91% unintentional vs. 71.89% intentional; *p* < 0.001). The majority of both unintentional or intentional poisoning patients were treated, evaluated, and released from the ED, with this outcome more frequently observed among the unintentional poisoning group (38.35% intentional vs. 50% unintentional; *p* < 0.001). On the other hand, intentional poisoning cases were more frequently admitted to the intensive care units (12.05% intentional vs. 7.99% unintentional; *p* = 0.048) as well as to a psychiatric unit 17.67% intentional vs. 11.6% unintentional; *p* = 0.012).

**Table 3:** Associations between ED management and disposition with intentional and unintentional poisoning (*n* = 886).

Variables		Intentional poisoning (*n* = 498, 56.2%)	Unintentional poisoning (*n* = 388, 43.8%)	*p*-value
**Length of time between exposure and ED presentation (hours)**	**Mean ± SD**	5.1 ± 14.6	2.8 ± 3.7	**0.002**
	**Median (IQR)**	1.8 (0.6- 4.3)	1.3 (0.7–3)	
**Length of stay in the ED (hours)**	**Mean ± SD**	11.3 ± 11.3	4.3 ± 5.2	**<0.001**
	**Median (IQR)**	6.9 (3.9–13.8)	3 (1.7–5.3)	
**Decontamination, yes**		95 (19.1%)	0 (0.0%)	**<0.001**
Undressing and skin decontamination		4 (0.8%)	0 (0.0%)	0.14
Activated charcoal (single/multiple dose)		78 (15.7%)	0 (0.0%)	**<0.001**
Gastric lavage		11 (2.2%)	0 (0.0%)	**0.003**
Whole bowel irrigation		5 (1.0%)	0 (0.0%)	0.07
**Antidotes, yes**		140 (28.1%)	12 (3.1%)	**<0.001**
**Charcoal**		44 (8.8%)	0 (0.0%)	**<0.001**
**Mucomyst (NAC)**		28 (5.6%)	12 (3.1%)	0.07
Other*		72 (14.5%)	0 (0.0%)	<0.001
**ED disposition**				
Left ED against medical advice		102 (20.5%)	77 (19.8%)	0.81
Treated/evaluated and released		191 (38.4%)	194 (50.0%)	**<0.001**
Admitted to a critical care unit (CCU)		60 (12.0%)	31 (8.0%)	**0.048**
Admitted to a psychiatric bed facility		88 (17.7%)	45 (11.6%)	**0.01**
Admitted to a non-critical care unit (NCCU)		30 (6.0%)	23 (5.9%)	0.95
Deceased		3 (0.6%)	0 (0.0%)	0.26
Transferred/Unknown disposition		24 (4.8%)	18 (4.6%)	0.90

* Bicarbonate; Antivenom; Benztropine; Pyridoxine; Glucagon; Atropine; Benzodiazepine; Thiamine; Naloxone; Glucose.

As for the patients who left the ED against medical advice, 20.48% were among the intentional group versus 19.85% in the unintentional group. However, this difference was not statistically significant (*p* = 0.815).

The final hospital outcome of patients is presented in Table 4. No effects were more frequently observed in the unintentional poisoning group than the intentional poisoning group (33.13% intentional vs. 42.53% unintentional; *p* = 0.004). On the other hand, most intentional poisoning cases resulted in minor effects after substance exposure (46.18% intentional vs. 37.11% unintentional; *p* = 0.007). To note, both moderate and severe outcomes are as common in unintentional poisonings as in intentional ones.

**Table 4:** Associations between medical outcome of exposed patients with intentional and unintentional poisoning (*n* = 886).

Variables	Intentional poisoning (*n* = 498, 56.2%)	Unintentional poisoning (*n* = 388, 43.8%)	*p*-value
**No effect**	165 (33.1%)	165 (42.5%)	**0.004**
**Minor effect**	230 (46.2%)	144 (37.1%)	**0.007**
**Moderate effect**	67 (13.5%)	57 (14.7%)	0.60
**Major effect**	18 (3.6%)	8 (2.1%)	0.17
**Death**	3 (0.6%)	0 (0.0%)	0.26
Other*	15 (3.0%)	14 (3.6%)	0.62

* Unrelated effect, the exposure was probably not responsible for the effect(s); Patient left before completion of care with no follow-up, minor effect expected; Patient left before completion of care with no follow-up, major effect expected; unknown.

The logistic regression model identified several factors significantly associated with an increased likelihood of intentional exposures (Table 5). Compared to males, females demonstrated a 1.82-fold higher risk (95% CI: 1.31–2.52, *p* < 0.001). Furthermore, each 10-year increase in age was associated with a 1.29-fold increase in odds. of intentional poisoning (95% CI: 1.18–1.40, *p* < 0.001). Individuals diagnosed with mood disorders exhibited a markedly increased risk, with odds ratios reaching 22.63 (95% CI: 11.67–43.86, *p* < 0.001). Similarly, those with neurotic, stress-related, or somatoform disorders showed significantly higher odds of intentional poisoning (OR: 3.71, 95% CI: 1.99–6.93, *p* < 0.001). While schizophrenia/schizotypal/delusional disorders did not show a significant association (*p* = 0.36), individuals with behavioral and emotional disorders originating in childhood or adolescence exhibited a substantially increased risk (OR: 10.93, 95% CI: 2.34–51.13, *p* = 0.002).

**Table 5:** Multivariate logistic regression analysis predicting intentional poisoning (*n* = 886).

Intentional poisoning (reference: unintentional poisoning)		
Variables	OR (95% CI)	*p*-value
**Females**	1.82 (1.31 - 2.52)	**<0.001**
**Age, years**	1.29 (1.18 - 1.40)	**<0.001**
**Mood disorders**	22.63 (11.67 - 43.86)	**<0.001**
**Neurotic/stress-related/somatoform disorders**	3.71 (1.99 - 6.93)	**<0.001**
**Schizophrenia/schizotypal/delusional disorder**	2.30 (0.39 - 13.46)	0.36
**Behavioral and emotional disorders with onset usually occurring in childhood and adolescence**	10.93 (2.34 - 51.13)	**0.002**

Variables included in the model: Age (increased by 10 years); gender (reference: male); Mood disorders; Neurotic/stress-related/somatoform disorders; Schizophrenia/schizotypal/delusional disorder; Behavioral and emotional disorders with onset usually occurring in childhood and adolescence.

## Discussion

This study provides a descriptive comparison of all intentional and unintentional poisoning cases that presented to the emergency department at a tertiary care center in Lebanon, encompassing a total of 886 cases.

Our findings indicated a higher incidence of intentional poisoning compared to unintentional poisoning. Females were more frequently involved in intentional poisoning, which aligns with findings from other countries such as the US and Qatar [9,10]. In contrast, no significant gender disparity was observed in the unintentional group. This differs from findings in Pakistan, where men are more prone to both intentional and unintentional poisoning [11].

In Lebanon, most unintentional toxicological exposures occur among young adolescents and children under the age of six. This trend is consistent with data from many other countries, including the US, United Arab Emirates, Jordan, Saudi Arabia, and many other countries, where the high incidence may be attributed to children’s curiosity and exploratory behaviors [2,12–14]. In addition, the lack of clear policies regarding childproof storage in Lebanon, along with the improper safekeeping and labeling of drugs, further exacerbates poisoning cases among young children.

In our study, most poisoning cases among individuals aged 20–50 years were intentional. However, in the US, unintentional exposures outnumber intentional poisoning cases in all age groups, except those aged between 13 and 19 years, though the majority of fatal intentional poisoning cases were reported among individuals aged 20 years and older [2].

Sedatives, hypnotics, and antipsychotics group were the most used drugs in poisoning cases, with benzodiazepines, particularly alprazolam, being highly prevalent. This finding aligns with a Lebanese study by El Majzoub et. al. (2018), which showed that benzodiazepines were the most used drugs, accounting for 39.3% of cases, based on data analysis from 2009 till 2015 [15]. Another study done by El Zahran et. al (2022), demonstrated that there is a high rate of benzodiazepine use in Lebanon among which 64% were found to be females and young adults aged 18–40 years [16]. Unlike the US, where sedatives rank sixth in human exposures [2], their predominance in Lebanon highlights weak prescription monitoring and the need for stricter regulation [16]. While Lebanon does not face an opioid epidemic like the US, benzodiazepines misuse remains a significant concern due to its potential for dependency and abuse [16,17]. Given the high prevalence of psychiatric disorders, addressing this issue is crucial to safeguarding patients’ mental health and preventing a benzodiazepine use disorder.

Analgesics, particularly acetaminophen, are the second most common exposure agents involved in both intentional and unintentional poisoning cases, which aligns with trends in multiple countries where analgesics occupy the first rank in the list [2]. In Lebanon, just like most of the Middle East, paracetamol can be purchased over the counter without any restriction on the quantity, which likely explains the high numbers of analgesic poisoning cases [18]. Antidepressants on the other hand rank third in Lebanon. In a study done in Bekaa, Lebanon, 61.1% of the residents that were interviewed took antidepressants in the past 3 months [19].

Concerning the unintentional poisoning cases, 5.7% of them were due to pesticide poisoning. Pesticide poisoning poses a significant public health problem in many countries other than Lebanon. For example, in the US, strict regulations and public education decreased the pesticide poisoning cases incidence by 61% from 1998 to 2020 [20]. Implementing public health awareness and comprehensive efforts by healthcare workers, pesticide manufacturers contributed to this decline. Similarly, in India, strict policies have been implemented on highly hazardous pesticides used for agriculture, which led to the decline of total suicide numbers and poisonings [21]. In contrast, this issue is still present in Lebanon due to the inadequate public awareness, lack of regulations, improper labeling, and unsafe storage. Therefore, to achieve a similar success, public awareness campaigns on safe use and storage in high-locked cabinets for example and strict regulatory legislations should be implemented. Similarly, household cleaning substances accounted for 7.47% of poisonings, reflecting improper labeling and storage. This underscores the essential problem of improper labeling, unsafe storage, and handling of cleaning substances in households. A study conducted by El Zahran, et. al in 2022, accentuated this improper storage and labeling of cleaning substances among consumers in Lebanon and the need to implement educational campaigns to improve their handling [22].

It is also important to note that salbutamol inhalers are contributing to unintentional poisoning cases in 1.03% in Lebanon and approximately 14.3% of cases between 2009 and 2017 in the US [23]. Keeping in mind that the unintentional poisoning cases mostly occur in children aged less than 6 years, it is crucial to prevent these salbutamol poisoning cases. The salbutamol drug lacks childproof packaging in Lebanon. The Poison Prevention Packaging Act has played an instrumental role in protecting children from many hazardous incidents by implementing child-resistant packaging forms [24]. Given the persistence of salbutamol poisonings among children, it is important to extend this act to include these inhalers. Applying a secured child-lock on these inhalers could significantly prevent these incidents from happening to these vulnerable populations. Unintentional poisoning cases occur predominantly in children under six, with moderate and severe outcomes as frequent as those in intentional cases. This underscores the need to promptly identify unintentional poisonings to prevent harm to this vulnerable group. The potential severity of outcomes stresses the importance of preventative measures such as secure storage, clear labeling, and child-resistant packaging for medications like salbutamol.

Lastly, our analysis identified key predictors of intentional poisoning cases which were statistically significant, such as female gender (OR=1.82, *p* < 0.001), older age (OR=1.29 per 10-year increase, *p* < 0.001), and psychiatric conditions such as mood disorders (OR = 22.63, *p* < 0.001) and neurotic/stress-related/somatoform disorders (OR = 3.71, *p* < 0.001). Females and patients of older age are more likely to engage in intentional poisoning compared to others. Similarly, most studies done in the Eastern Mediterranean Region Countries (like in Qatar, Egypt, and Yemen) showed that there is a higher prevalence of intentional poisoning in females than males [10,25,26]. A high odds ratio of 22.63 was found specifically for mood disorders, indicating that patients with these disorders are around 22 times more prone to commit intentional poisoning than those without such diagnoses. A wide confidence interval with an odds ratio of 10.93 was found for behavioural and emotional disorders with onset occurring in childhood and adolescence and although significant, it might reflect a small sample size in this category in specific. These significant findings highlight the need for psychiatric support needed among the Lebanese population to try to prevent these events from happening in the first place. However, this significance was not seen among schizophrenia/schizotypal/delusional disorders (*p* = 0.36).

Beyond its clinical relevance, our study carries important operational and policy implications for both emergency care and public health in Lebanon. Within the emergency department, the predominance of intentional poisonings among young adults—particularly women and patients with psychiatric comorbidities—highlights the need for standardized triage protocols that incorporate early psychiatric screening and referral pathways. Given the high prevalence of benzodiazepine-related poisonings, especially alprazolam, emergency physicians should also be equipped with clear management algorithms for sedative overdoses. At the community level, the large burden of unintentional poisonings among children under six underscores the urgent need for poison prevention measures such as child-resistant packaging, improved household education on safe storage, and clear labeling of both medications and cleaning substances. The recurrent involvement of readily available agents such as salbutamol inhalers, further emphasizes the importance of regulating dispensing practices and packaging requirements. More broadly, these findings call for a coordinated national strategy in Lebanon that links ED-based toxicology data to a national surveillance system. Such an approach could enable policymakers to monitor emerging trends in poisoning, guide restrictions on high-risk substances, and design targeted interventions for vulnerable populations.

This study has several limitations. First, due to the lack of a national poison centre in Lebanon, a notable limitation lies in most of the reporting being single centred to the clinical toxicology service data. This may especially limit the ability to generalize the findings to other healthcare setting or other populations due to the differences in population demographics, healthcare facilities and management practices. This highlights the importance of establishing a national poison center that would be more representative of the whole country and that would include calls from the public in addition to calls from healthcare providers. Second, the retrospective nature of this study may introduce many biases such as selection bias, recall bias, incomplete or inconsistent documentation in the medical records. The use of pre-existing data carries the risk of missing or imprecise documentation for some variables. However, this has been accounted for through extensive data extraction, cleaning, and application of statistical methods to adjust for any confounder. Third, there is a risk of misclassification bias when determining whether the poisoning was intentional or unintentional and even its subcategories because this is based on clinician documentation and patient’s subjective self-report. Finally, common to all observational studies, unmeasured confounding variables can also impact the exposure and outcome of the study.

## Conclusion

In conclusion, this study shows that intentional poisonings (56.21%) are more common than unintentional ones (43.79%) in Lebanon, with sedatives, hypnotics, and antipsychotics being the most used substances. Poisoning trends in Lebanon differ from the US, particularly in sedatives, pesticides, and salbutamol inhalers, but are similar in household cleaning products and the higher incidence of unintentional poisonings in the under-18 group. We recommend improved screening of poisoning cases in ED visits and better prevention through stricter regulation of medications and safe storage of hazardous materials like pesticides.

## Supporting information

S1 File Dataset. Intentional-unintentional poisoning dataset.
(SAV)s
